# Sudden unexpected death in a 17-year-old boy due to unacknowledged adamantinoma-like Ewing sarcoma

**DOI:** 10.1007/s12024-022-00525-x

**Published:** 2022-09-21

**Authors:** N. Pigaiani, F. Ausania, M. Tudini, F. Bortolotti, F. Tagliaro, M. Brunelli

**Affiliations:** 1 Unit of Forensic Medicine, Department of Diagnostics and Public Health, University of Verona, Piazzale L.A. Scuro 10-37134, Verona, Italy; 2 Bologna, Italy; 3 Unit of Pathology, Department of Diagnostics and Public Health, University of Verona, Piazzale L.A. Scuro 10-37134, Verona, Italy

**Keywords:** Adamantinoma-like Ewing sarcoma, Sudden unexpected death, Airway occlusion, Forensic pathology

## Abstract

A 17-year-old male with no previous medical history was admitted 2 days before his death to a local hospital after mild dyspnea. Electrocardiography, chest radiography, and blood analysis revealed no abnormalities. Blood oxygen saturation was 99%, and SARS-CoV-2 nasopharyngeal swabs tested negative; thus, he was discharged without prescriptions. After 2 days, the subject died suddenly during a pool party. Forensic autopsy was performed analyzing all anatomical districts. Cardiac causes were fully excluded after deep macroscopic and microscopic evaluation; lung and brain analyses showed no macroscopic pathology. Finally, a large subglottic solid mass was detected. The whitish neoplasm showed an aggressive invasion pattern to the thyroid and adjacent deep soft tissues and occluded the trachea. High-power microscopy showed sheets of small, uniform cells with scant cytoplasm; round nuclei; and small, punctate nucleoli, with immunohistochemical expression of CK8-18, AE1/AE3, and CD99. Using FISH analysis, the break-apart molecular probes (EWSR1 (22q12) Break – XL, Leica Biosystem, Nussloch, Germany) showed distinct broken red and green fluorochromes, diagnostic of Ewing sarcoma. The neoplasm was characterized as adamantinoma-like Ewing sarcoma, and the mechanism of death was identified as airway obstruction. The rarity of the case resides in the circumstances of death, which pointed to the possibility of sudden unexpected death due to heart disease, but an oncological cause and the underlying mechanism were finally diagnosed. The best method to perform autopsies is still complete, extensive, and systematic macroscopic sampling of organs and districts followed by histopathological analysis, in addition to immunohistochemical and molecular investigations in those cases in which they are necessary. In fact, when neoplasms are detected, the application of advanced techniques such as immunohistochemistry and molecular diagnostics is fundamental to accurately certify death.

## Case report

A 17-year-old white male with no previous abnormal medical history was admitted 2 days before his death to a local hospital after mild dyspnea arising a few days earlier. Electrocardiography, chest radiography, and blood analysis revealed no abnormalities. Blood oxygen saturation was 99%, and SARS-CoV-2 nasopharyngeal swabs tested negative; thus, he was discharged without prescriptions.

Two days later, the subject was attending a pool party. After diving into the pool, the patient complained of illness and severe dyspnea. Friends helped him to get out of the water, and suddenly, the young man became unresponsive. Emergency personnel immediately intervened. Emergency electrocardiography detected pulseless electrical activity (PEA), and cardiopulmonary resuscitation according to the ACLS protocol was initiated. Unfortunately, the heart rhythm became asystolic, and after 1 h of cardiopulmonary resuscitation, the young man died.

## Forensic autopsy

Forensic autopsy was performed 48 h later. The cadaveric inspection of the body (height 170 cm; weight 70 kg; body mass index 24.2) showed no pathologic data apart from venipuncture at the upper limb, evidence of the recent resuscitation attempts.

The cadaveric section revealed bilateral pleural effusion (500 ml of citrine fluid in each pleural cavity); heavy, congested, and edematous lungs (1400 g overall); and subpleural petechiae. Macroscopic cardiac and cerebral pathologies were fully excluded.

An extensive forensic autopsy was performed in which all anatomical districts were analyzed (comprehensive method), and finally, a subglottic solid mass was detected. The trachea and larynx were smeared with mucous and foamy secretions.

**Fig. 1:**
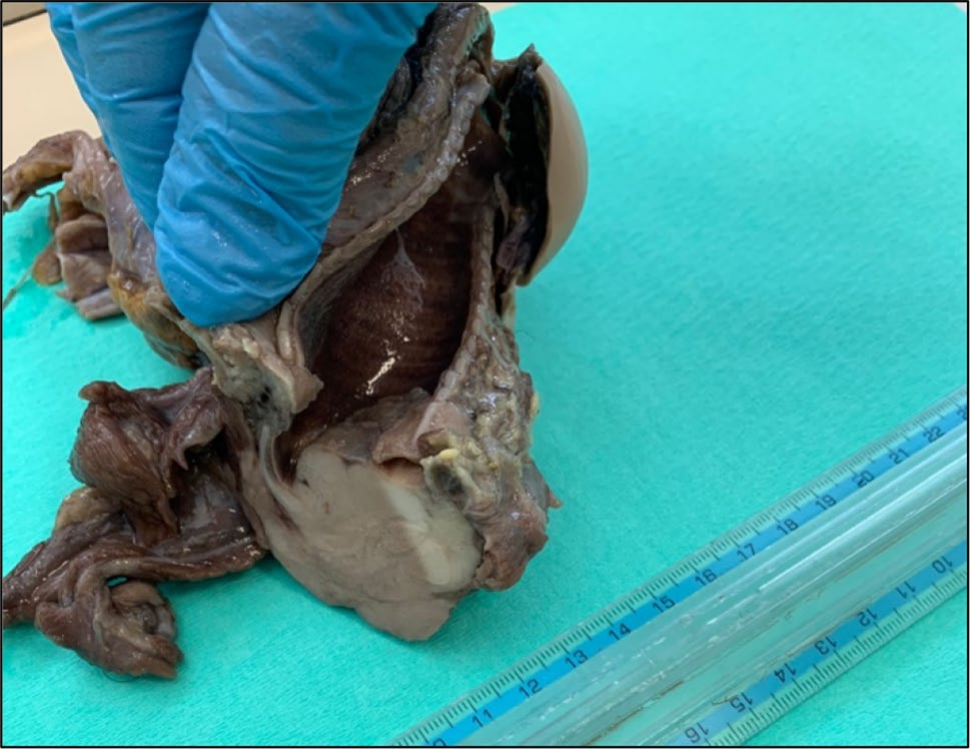
Macroscopical view of subglottic mass

**Fig. 2:**
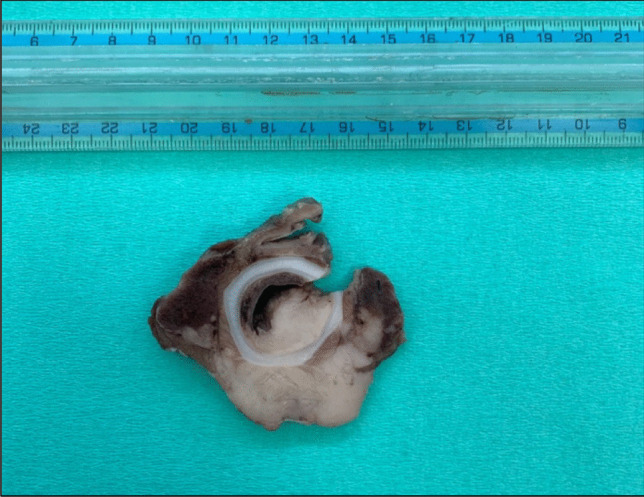
Transversal section of the subglottic mass

After a suitable formaldehyde fixation period, the larynx, trachea, and bronchi were examined. The whitish neoplasm showed an aggressive invasion pattern to the thyroid and adjacent deep soft tissues and occluded the trachea (Figs. 1 and 2).

## Toxicological analysis

Comprehensive immunoenzymatic screening (CEDIA®) was performed on urine and vitreous humor after sample centrifugation. No common drugs of abuse were identified. A general toxicological screening for therapeutic and illicit drugs was also performed on the autopsy blood samples using a Toxtyper LC/Ion Trap-MS platform (Bruker Daltonics, Bremen, Germany) with a recently published method [1]. No drugs of abuse or medicinal compounds were identified on the basis of the Maurer-Wissenbach-Weber MS spectrum libraries.

Blood alcohol concentration was determined with head-space gas chromatography coupled with a flame-ionization detector (HS-GC-FID), and the results were below the lower level of quantification (LLOQ = 0.05 g/l).

## Histology and molecular investigation

**Fig. 3:**
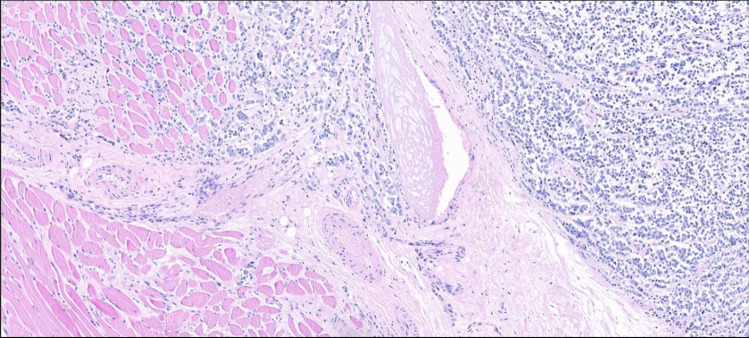
Small cells with slight cytoplasm, round nuclei, and small punctate nucleoli with invasive pattern to muscular tissues (hematoxylin and eosin stain; × 40 magnification)

**Fig. 4:**
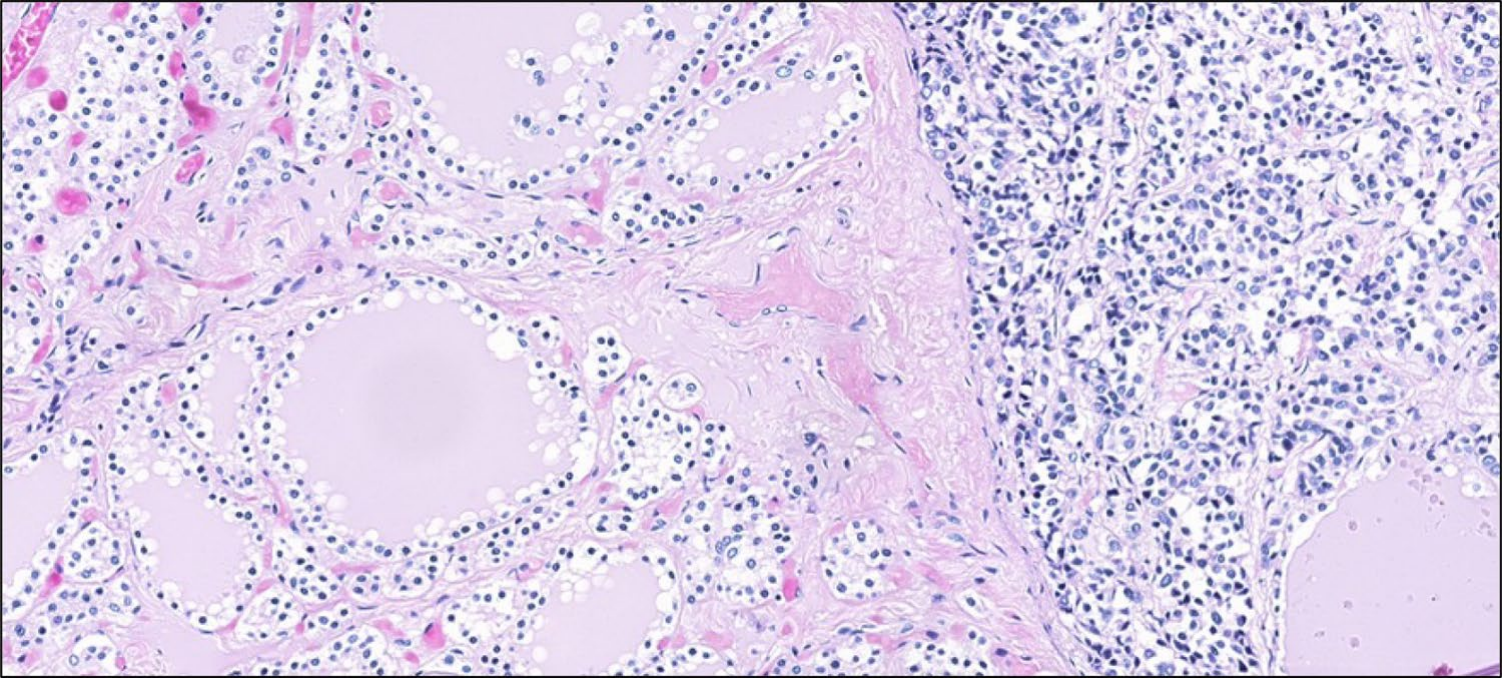
Small cells with slight cytoplasm, round nuclei, and small punctate nucleoli with invasive pattern to thyroid parenchyma. A thyroid follicle enclosed by tumor cells is visible in the lower right part of the figure (hematoxylin and eosin stain; × 80 magnification)

**Fig. 5:**
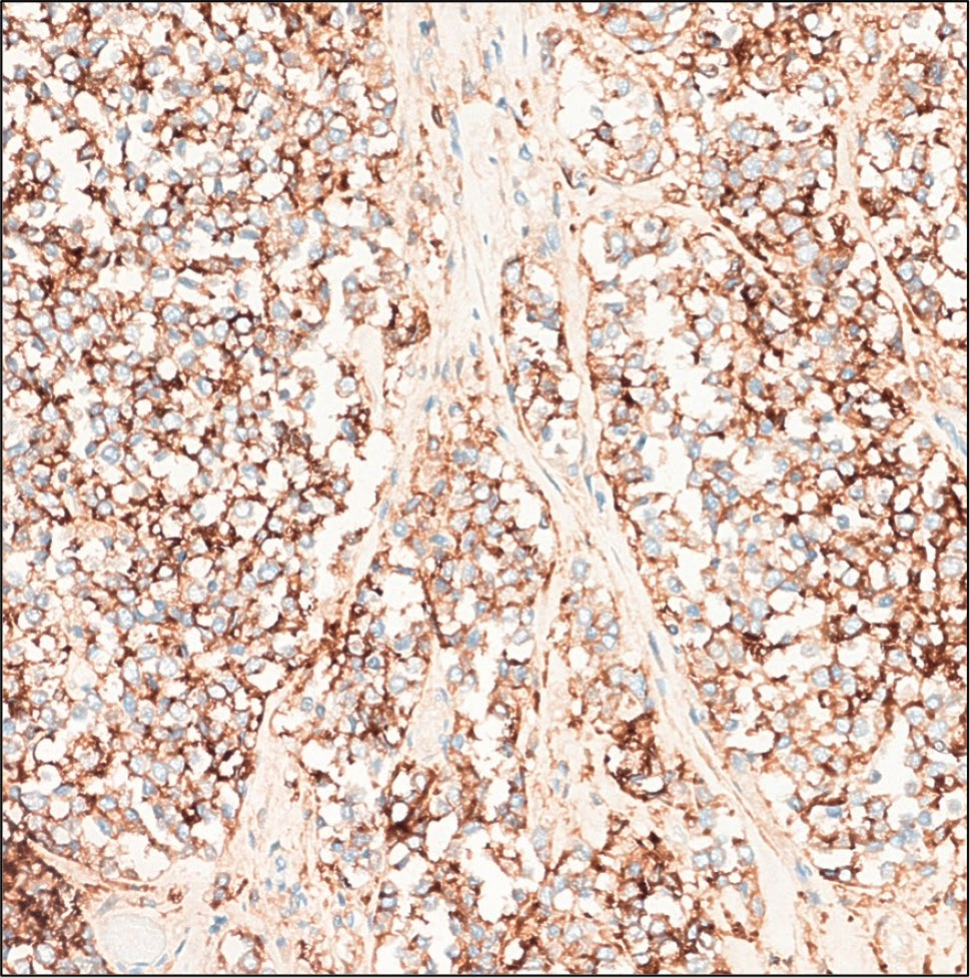
Immunohistochemical expression of CD99 (× 80 magnification)

High-power microscopy showed sheets of small, uniform cells with scant cytoplasm; round nuclei; and small, punctate nucleoli (Figs. 3 and 4) with a pattern of invasion to the muscular tissues of the neck and thyroid parenchyma. Immunohistochemistry analysis revealed the immunoexpression of CK8-18 and AE1/AE3 and CD99 (Fig. 5). Thyroglobulin, TTF1, PAX8, calcitonin, CK20, SOX10, ERG, SMA, and NUT were all negative at the immunophenotype level.

Given the previous evidence, molecular analysis was then carried out.

**Fig. 6:**
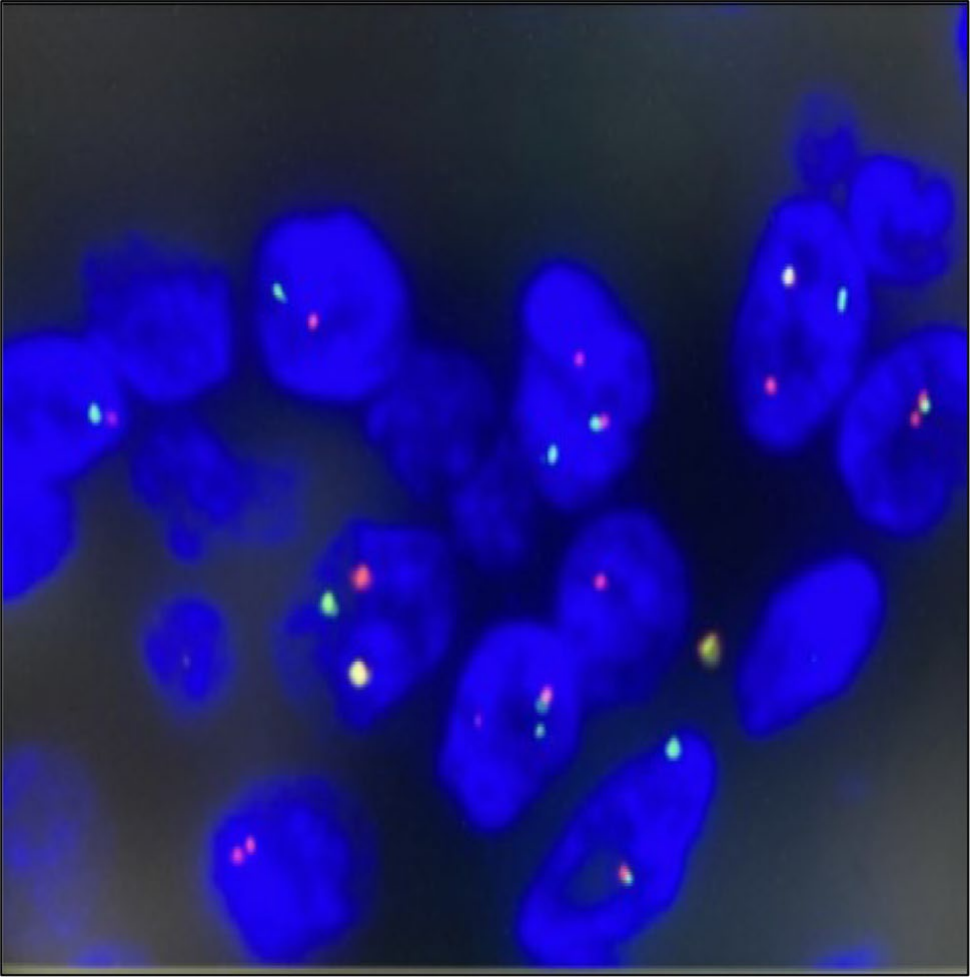
FISH. Separation of green and red fluorescent signals, sign of rearrangement in the EWSR1 locus

Using FISH analysis, the break-apart molecular probes (EWSR1 (22q12) Break – XL, Leica Biosystem, Nussloch, Germany) showed distinct broken red and green fluorochromes (Fig. 6), diagnostic of Ewing sarcoma.

The neoplasm, potentially characterizable as an undifferentiated carcinoma of the neck by classic histological examination alone, was finally typed as adamantinoma-like Ewing sarcoma (ALES) thanks to immunohistochemistry and molecular biology analyses. The cause of death was identified as airway obstruction resulting from subglottic ALES.

## Discussion

Sudden unexpected deaths due to undiagnosed neoplastic disease in infancy and childhood are rare, with reported incidences ranging from 0.10 to 0.47% [2, 3]. The vast majority of these deaths are caused by unrecognized malignancies that affect critical systems, such as the central nervous and cardiovascular systems. In addition, several undiagnosed hematologic neoplasms have been reported [4–6].

In particular, many cases of sudden death in young people due to unidentified neoplasms affecting the central nervous system have been reported in the literature. Histotypes included but were not limited to meningioma, astrocytoma, oligodendroglioma, glioblastoma, ependymoma, germinoma, teratoma, and adenoma [7–11]. The mechanisms of sudden death due to intracranial tumors are heterogeneous and can be attributed to the increase in intracranial pressure, direct compression of vital nervous centers, spontaneous intratumor hemorrhage, or brain invasion [12].

In cases of sudden death in young patients due to cardiac neoplasms, literature reports include hemangioma, sarcoma, and inflammatory pseudotumor cases. In these events, death occurred from fatal arrhythmias or the obstruction of coronary arteries [13–16].

Literature about neoplasms affecting the respiratory system that are responsible for sudden death in infancy and childhood is scarce. In the reported cases, the terminal mechanism ranges from external compression to mass forming processes obstructing airways [17, 18].

In the case reported herein, it is possible to assume that the presentation of dyspnea in the days before death may have indicated the reaching of a critical tumor mass that impeded proper airflow. During the pool party, the subject took a dip in the pool before complaining of severe dyspnea and subsequently lost consciousness. The authors’ opinion is that the contact between the pool water containing irritants such as chlorine and the upper airway, the site of the stenosing tumor, caused an irritative reaction; the resulting mucus secretion led to the complete obstruction of the upper airway, resulting in the subject’s death by asphyxiation. These considerations are consistent with the evidence of pleural petechiae and heavy and congested lungs, nonspecific but suggestive signs of asphyxial death.

Due to the rarity of the clinical presentation (i.e., death) and the impossibility of making the diagnosis on the basis of only histological evidence, genome-wide and immunophenotype analyses were necessary. The tumor showed the expression of cytokeratins, such as CK8-18 and AE1/AE3, and typical CD99 expression, whereas immunostaining for thyroglobulin, TTF1, PAX8, calcitonin, CK20, SOX10, ERG, SMA, and NUT was negative.

FISH analysis of formalin-fixed and paraffin-embedded tissues is routinely used worldwide to define EWRS1 gene rearrangements such as translocation. Although EWSR1-FLI1 gene fusion was traditionally considered pathognomonic for the diagnosis of Ewing sarcoma, it is well known that many partners other than FLI1 are identifiable [19]. Indeed, investigations for the wrong fusion partners would give a negative result. For these reasons, break-apart probes rather than knowledge of the partner locus are needed to establish the EWRS1 rearrangement, making break-apart probes the recommended FISH kit for EWRS1 rearrangement diagnostics. In fact, RT-PCR analysis may provide knowledge of the fusion product, information that is not necessary for the interpretation of EWRS1 rearrangements [19, 20].

In particular, locus-specific EWSR1 (22q12) break probes detect genomic translocations related to the EWSR1 gene in formalin-fixed and paraffin-embedded tissues. The probes have been manufactured to identify regions proximal and distal to the breakpoints in the EWSR1 locus. Both probes are designed to detect translocations of the EWSR1 gene at 22q12 by fluorescent signals. The separation of fluorescent signals is evidence of gene rearrangement. The chimeric protein dysregulates target genes, leading to oncogenic transformation, and is required for tumorigenesis in Ewing sarcoma. Different translocation partners are known, with similar overall survival [20].

According to the reported histology, immunohistochemistry evidence, the immunoexpression of high molecular weight cytokeratins, and the demonstration of EWSR1 gene rearrangement, the neoplasm was typed as ALES.

ALES was described by Bridge et al. in 1999 [21]. The neoplasm is a subset of Ewing sarcoma showing hybrid features of adamantinoma of the bones and Ewing sarcoma. In addition to harboring the t(11;22) translocation and EWSR1-FLI1 gene fusion traditionally reported as pathognomonic for a diagnosis of Ewing sarcoma, this tumor showed epithelial differentiation, mainly the immunohistochemical expression of p40 and cytokeratins. Although ALES was first recognized in the thorax and limbs, this histotype has been predominantly described in the head and neck district. Regarding the neck district, the neoplasm has presented as painless neck masses [22]. In addition, a recent review of ALES of the thyroid identified the age range of affected patients as 19–42 years (mean: 29.5), reporting good outcomes after total thyroidectomy and Ewing sarcoma therapy protocol, radiation, or radioactive iodine [23].

To the best of our knowledge, no case of asphyxial death caused by airway obstruction due to ALES has been reported thus far. The rarity of the reported case lies in the circumstances of death, which pointed to the probable sudden unexpected death of the young man due to heart disease (no significant medical history or sudden presentation), but the oncological cause and pathogenesis were finally diagnosed.

The best method to perform forensic autopsies is still the complete, systematic, and extensive macroscopic and microscopic investigation of all organs and districts. In addition, wide-ranging immunohistochemical and molecular analyses could be necessary in cases where a mass-forming process is recognized as the terminal mechanism of sudden unexpected death to accurately certify the death.
